# A Randomized Controlled Trial of Auricular Transcutaneous Electrical Nerve Stimulation for Managing Posthysterectomy Pain

**DOI:** 10.1155/2011/276769

**Published:** 2011-06-09

**Authors:** Hin Cheung Tsang, Chi Shan Lam, Ping Wing Chu, Jacqueline Yap, Tak Yuen Fung, Gladys L. Y. Cheing

**Affiliations:** ^1^Department of Rehabilitation Sciences, The Hong Kong Polytechnic University, Hung Hom, Kowloon, Hong Kong; ^2^Department of Physiotherapy, North District Hospital, Sheung Shui, Hong Kong; ^3^Department of Anaesthesiology and Operating Services, North District Hospital, Sheung Shui, Hong Kong; ^4^Department of Obstetrics and Gynaecology, North District Hospital, Hong Kong

## Abstract

*Background*. A patient- and assessor-blinded randomized controlled trial was conducted to examine the effectiveness of auricular transcutaneous electrical nerve stimulation (TENS) in relieving posthysterectomy pain. *Method*. Forty-eight women who had undergone a total abdominal hysterectomy were randomly assigned into three groups (*n* = 16 each) to receive either (i) auricular TENS to therapeutic points (the true TENS group), (ii) auricular TENS to inappropriate points (the sham TENS group), or (iii) 20 minutes of bed rest with no stimulation (the control group). The intervention was delivered about 24 hours after the operation. A visual analogue scale was used to assess pain while resting (VAS-rest) and upon huffing (VAS-huff) and coughing (VAS-cough), and the peak expiratory flow rate (PEFR) was assessed before and at 0, 15, and 30 minutes after the intervention. *Result*. As compared to the baseline, only the true TENS group reported a significant reduction in VAS-rest (*P* = .001), VAS-huff (*P* = .004), and VAS-cough (*P* = .001), while no significant reduction in any of the VAS scores was seen in the sham TENS group (all *P* > .05). In contrast, a small rising trend was observed in the VAS-rest and VAS-huff scores of the control group, while the VAS-cough score remained largely unchanged during the period of the study. A between-group comparison revealed that all three VAS scores of the true TENS group were significantly lower than those of the control group at 15 and 30 minutes after the intervention (all *P* < .02). No significant between-group difference was observed in PEFR at any point in time. *Conclusion*. A single session of auricular TENS applied at specific therapeutic points significantly reduced resting (VAS-rest) and movement-evoked pain (VAS-huff, VAS-cough), and the effects lasted for at least 30 minutes after the stimulation. The analgesic effects of auricular TENS appeared to be point specific and could not be attributed to the placebo effect alone. However, auricular TENS did not produce any significant improvement in the performance of PEFR.

## 1. Introduction

Postoperative pain is the most common form of acute pain [1]. In 2005, it was estimated that approximately 45 million patients in the US were suffering from postoperative pain [2]. Despite advancements in analgesic drugs and techniques, the management of postoperative pain remains a challenge for medical practitioners [3]. Clinical management for moderate to severe postoperative pain mainly involves the use of opioids. However, dose-related adverse effects have limited their use [4, 5], and it has been suggested that opioids are only effective in relieving resting pain rather than the pain associated with physical activities (e.g., coughing, mobilization) [4, 6–8].

Given the inherent side effects and limitations of conventional opioid analgesics, studies have been conducted to explore the effective use of nonopioids or even non-pharmacological analgesic techniques. Transcutaneous electrical nerve stimulation (TENS) is a non-pharmacological treatment modality for managing postoperative pain. Conventional use of TENS for managing postoperative pain usually applied the electrodes adjacent to the incision site. This may have increased the patient's chances of getting an infection. Interestingly, recent studies have found that the application of TENS on acupuncture points is also effective at producing analgesia [9, 10]. According to Traditional Chinese Medicine, acupuncture points exist not only on the body but also on the external ears [11], that is, the auricles. Previous studies have demonstrated that auricular TENS can raise the experimental pain threshold [12–15], decrease distal extremity pain [16], and reduce phantom limb pain [17] as well as the pain associated with burn wounds [18].

Various forms of auriculotherapy, such as auriculopressure and auricular acupuncture, have been found to be effective in managing pain after abdominal [19–24] and orthopedic surgeries [25, 26], but the effects of auricular TENS on controlling postoperative pain remain uncertain. Therefore, a patient- and assessor-blinded randomized controlled trial was conducted to examine the effectiveness of auricular TENS in controlling postoperative pain in patients after they have undergone a total abdominal hysterectomy. It was hypothesized that auricular TENS produces a greater analgesic effect than sham TENS or the control condition in patients after a total abdominal hysterectomy. The primary endpoint was the postoperative pain measured by Visual Analogue Scale (VAS). Specifically, the effectiveness of auricular TENS in reducing resting pain and movement-evoked pain (e.g., pain after huffing and coughing) was investigated. The secondary endpoint was the peak expiratory flow rate (PEFR).

## 2. Methods

Ethical approvals were obtained from the Hospital Authority of Hong Kong and the Hong Kong Polytechnic University. Sixty gynecological patients (ASA I–II) scheduled to undergo an elective total abdominal hysterectomy were recruited. Patients were excluded if they were above 60 years of age, suffered from pain or injuries in the ears, had an implanted cardiac pacemaker, a history of neurological disorders that could alter their perception of pain, prior experience with auriculotherapy, pulmonary disease or dysfunction, or renal failure, or were unable to give consent or follow instructions during the data collection process. They were also excluded if any unexpected event had occurred during or after the operation.

### 2.1. Experimental Procedures

Patients who fulfilled the study criteria were interviewed by the investigator before the operation, and the purposes of the study were explained to them. Their written consent to participate in the study was then obtained. The preoperation PEFR was recorded during the visit as the baseline measurement. Postoperative pain was assessed by the use of VAS. Patients were instructed to report the VAS score by putting a mark on a 10 cm horizontal line that had the words “no pain” at the left end and “pain as bad as it could be” at the other end. The PEFR was measured by using a pocket-sized spirometer (Spirodoc MIR-Medical International Research, Italy). All of the patients were told to perform a minimum of three trials for all of the PFER measurements during the study in order to meet the criteria for reproducibility as recommended by the American Thoracic Society [27]. One minute of rest was allowed between each trial. The best result was recorded for further analysis. 

All of the patients received an operation under a standardized general anesthesia protocol and no premedication was given. Anesthesia was induced with fentanyl (1-2 *μ*g/kg) and propofol (1-2 mg/kg) and was maintained with isoflurane (titrated to the patient's requirements) and nitrous oxide (60%) in oxygen (40%). Tracheal intubation was facilitated with cisatracurium (0.15–0.2 mg/kg). Morphine (0.1 mg/kg) was given as intraoperative analgesia. Residual neuromuscular blockade was reversed using neostigmine (0.05 mg/kg) and atropine (0.02 mg/kg) at the end of the operation. In the recovery room, postoperative pain was managed by an intravenous bolus of morphine (1-2 mg) as requested by the patient. Data including the type of incision (transverse or midline), duration of the operation and general anesthesia, the consumption of analgesics (during the operation and in the recovery room), and any complications that occurred during the operation were recorded. Postoperative analgesics were standardized with intramuscular pethidine injection (1 mg/kg, maximum 75 mg, once every 4 hours as requested) and dologesic tablet (containing 325 mg of acetaminophen and 50 mg of propoxyphene, once every 6 hours) when oral feeding was allowed.

All of the patients were assessed around 24 hours after the operation to determine whether there were any postoperative complications. Since the duration of action for pethidine lasts for about 2 to 3 hours [28] and dologesic about 2 to 4 hours [29], the time of the intervention was adjusted individually to ensure that no analgesic was given 3 hours prior to the study. This was to minimize the influence of pharmacological analgesia on the effects of auricular TENS.

Based on computer-generated codes, block randomization was used with a block size of 3 or 6. Patients were randomly assigned to one of the three groups: the true TENS group (*n* = 16), the sham TENS group (*n* = 16), and the control group (*n* = 16). Before the study began, the following information was recorded: the postoperative consumption of analgesics, the time of the last intake of analgesics, and the time of the intervention (number of hours after the operation). The interval between the last intake of analgesics and the intervention was calculated as the analgesic-free period for subsequent data analysis. The patients were all put in a half-sitting position on the bed and first asked to report their resting pain level (VAS-rest). The preintervention PEFR was measured, and the patients were then asked to report the worst pain that they had experienced during the PEFR measurements (VAS-huff). After a two-minute period of rest, the patients were asked to cough with maximal effort and the pain score (VAS-cough) was recorded. A blinded assessor who did not know the allocation of the groups was responsible for collecting all of the data.

The auricular TENS therapy was delivered by the investigator, who was a registered physiotherapist and had accredited for practicing acupuncture. Auricular TENS was delivered by using an electroacupuncture unit (IC-4107, ITO Ltd., Japan) with a biphasic pulse at a frequency that ranged from 1 to 300 Hz and a pulse width that ranged from 50 to 150 *μ*s. The electroacupuncture unit consisted of a hand-held probe with a diameter of 2 mm, which can be used to search for acupuncture points as well as to provide stimulation. Before the intervention, the patients were asked to remove any earrings and necklaces. The skin at all stimulation sites was cleaned with cotton gauze and alcohol to reduce skin resistance. The patients were asked to hold the dispersive electrode with one hand, while the locations of the auricular points were identified using the search probe. The appropriate locations of the auricular points were confirmed by the area of the lowest skin resistance through the audible signal coming from the built-in buzzer of the electroacupuncture unit.

**Figure 1:**
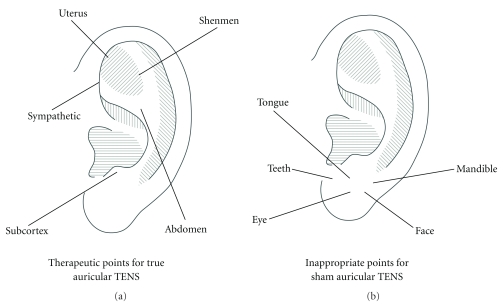
Auricular points chosen for stimulation.

According to the recommendation made in the literature [11, 30–32] and advice given by a registered practitioner of Traditional Chinese Medicine, the true TENS group received auricular TENS delivered to the following five therapeutic points: “uterus,” “abdomen,” “sympathetic,” “shenmen,” and “subcortex” (Figure 1). The sham TENS group received auricular TENS delivered to the following five inappropriate points: “teeth,” “tongue,” “mandible,” “eye,” and “face” (Figure 1). These inappropriate points are believed to have no analgesic effects after a total abdominal hysterectomy [11, 33]. Patients in the two treatment groups received electrical stimulation at auricular points on both ears (a total of 10 points). Patients in the true TENS group received auricular stimulation in the order of “uterus,” “abdomen,” “sympathetic,” “shenmen,” and “subcortex,” starting on the left side and repeated in the same order on the right side. For the sham TENS group, auricular points were stimulated in the order of “teeth,” “tongue,” “mandible,” “eye,” and “face,” also starting on the left side and then repeated on the right side. Each auricular point was stimulated at a frequency of 1 Hz for 90 seconds with an intensity of current up to the patients' maximum level of tolerance. Patients were instructed to respond verbally to the increasing intensity by saying “Feel it,” to indicate that they felt the electrical stimulus, and by saying “Stop” when they felt pain, at which time the intensity was not increased further. The treatment techniques and parameters were devised with reference to those used in previous studies [14, 18, 34]. Patients in the control group did not receive any auricular TENS and were instructed to rest in bed for 20 minutes. After the interventions, the VAS-rest, PEFR, VAS-huff, and VAS-cough were reassessed by the blinded assessor in the same sequence as in the preintervention assessment. A reassessment was performed immediately after the intervention and repeated 15 minutes and 30 minutes after the intervention.

### 2.2. Statistical Analysis

The patients' demographic profile and the baseline data were compared using one-way analysis of variance (ANOVA). The type of incision was analyzed using the Chi-square test. All postoperative PEFRs were expressed as a percentage of the baseline value. Repeated measures ANOVA were used to examine the group and time effects on the scores of VAS-rest, VAS-huff, VAS-cough, and PEFR, respectively. When there was significant interaction between “group” and “time,” subsequent analyses were performed divided by “group” and “time.” A Bonferroni correction was performed to adjust the level of significance due to multiple comparisons. Any statistically significant result was followed by a post-hoc analysis with Tukey's honestly significant difference test. All analyses of data were performed using the Statistical Package for the Social Sciences (Version 16). Alpha was set at 0.05 for all statistical tests.

## 3. Results

**Figure 2:**
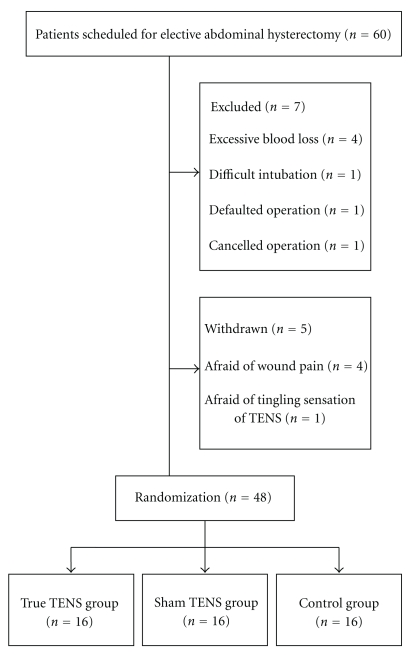
A triage profile of the study.

Among the sixty patients recruited before the operation, twelve were either excluded based on exclusion criteria or withdrew from the study after the operation. A consort diagram of the study is shown in Figure 2. Of the forty-eight patients who completed the study, there was no significant difference in any of the demographic data obtained at the baseline (all *P* > .05) (Table 1). None of the patients who received auricular TENS showed any complications or adverse reactions after the treatment.

### 3.1. The Changes in Resting Pain, Huffing Pain, and Coughing Pain over Time

**Figure 3:**
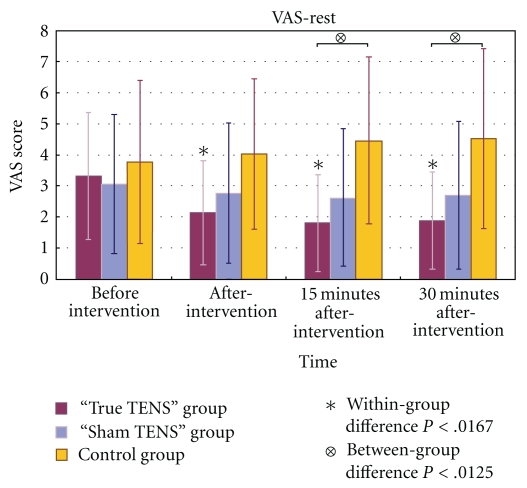
The mean VAS-rest scores of the three groups at different time points.

**Figure 4:**
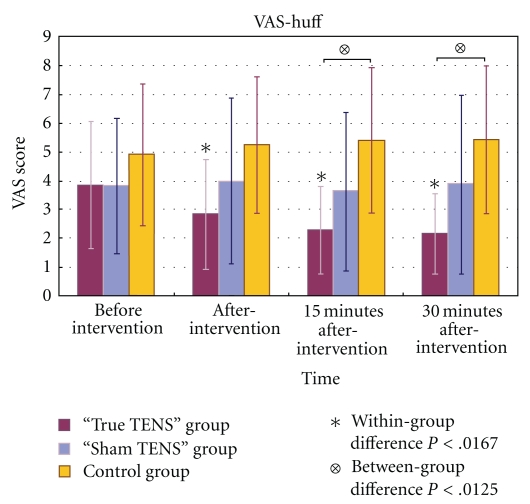
The mean VAS-huff scores of the three groups at different time points.

**Figure 5:**
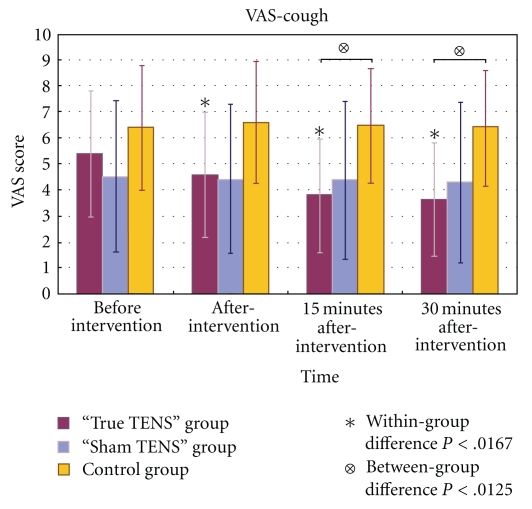
The mean VAS-cough scores of the three groups at different time points.

In the true TENS group, the mean scores for VAS-rest, VAS-huff, and VAS-cough decreased significantly over time (Figures 3, 4, and 5). The VAS scores of the true TENS group declined significantly at all time points after intervention as compared to the preintervention scores. The change was significant even after an adjustment was made to the *P* value by the Bonferroni correction (*P* = .05/number of tests; 0.05/3 = 0.0167). In contrast, no significant within-group difference was found in any of the three VAS scores in the sham TENS group and the control group.

Among the three VAS scores, there were significant between-group differences obtained immediately after the intervention and 15 and 30 minutes after the intervention. Significant group differences were maintained at 15 and 30 minutes after intervention, even after adjustment by the Bonferroni correction (*P* = .05/number of tests; 0.05/4 = 0.0125). A posthoc analysis with Tukey's test revealed that the differences mainly arose from the true TENS group and the control group (Figures 3–5).

### 3.2. The Changes in Peak Expiratory Flow Rate over Time

**Figure 6:**
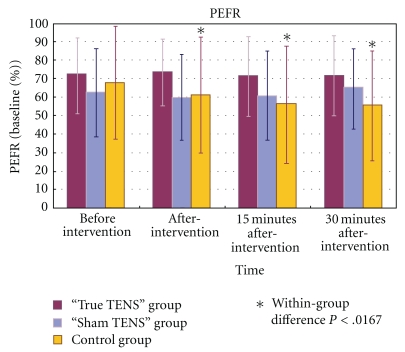
The mean PEFR of the three groups at different time points.

There were no significant changes in the PEFR within the true TENS group and the sham TENS group (Figure 6). In contrast, the control group showed a significant decrease in the PEFR over time. A post hoc test showed that the PEFR had decreased significantly at all time points after the intervention as compared to the baseline (*P* < .001).

As for the between-group comparisons, no significant differences in the PEFR were found among the three groups at all time points (all *P* > .05).

## 4. Discussion

We found that a single session of auricular TENS can significantly relieve the resting pain after a total abdominal hysterectomy. This finding is consistent with that of previous studies showing that auriculotherapy is effective in managing postoperative pain [19–26]. Usichenko et al. [25] demonstrated that auricular acupuncture significantly reduced the consumption of analgesics by patients after they had undergone a total hip arthroplasty and that auriculopressure [20, 22, 24] can be used to manage pain after abdominal surgeries.

Similar to acupuncture, auricular TENS produces gradual and progressive analgesia, implying that endogenous opioids may be involved [35]. Both body and auricular acupunctures have been found to raise the levels of endorphins and enkephalins in blood serum and cerebrospinal fluid [11], and auriculotherapy can increase beta-endorphin in plasma [36]. Simmons and Oleson [15] also found that analgesia induced by auricular TENS can be reversed by naxolone.

Our present findings illustrate that auricular TENS applied to therapeutic points can also reduce movement-evoked pain after the operation. This effect can be partly explained by the action of endogenous opioids, even though other mechanisms may also be involved. Rakel and Frantz [37] reported that TENS significantly reduced movement-evoked pain in people after abdominal surgery. They suggested that TENS may reduce the primary mechanical hyperalgesia that corresponds to a decrease in movement-evoked pain. Primary hyperalgesia enhances responses to mechanical and thermal stimuli in the area of incision. This is likely caused by the sensitization of primary afferent fibers [38]. A*δ* and C fiber nociceptors are usually sensitized by the prolonged presence of signaling molecules that signify “damage” or “inflammation” after an incision. These signaling molecules include substance P, serotonin, bradykinin, epinephrine, adenosine, and nerve growth factor [39]. The resultant conversion of mechanically insensitive silent A*δ* nociceptors to mechanically active fibers may account for the development of hyperalgesia after operation [40]. Secondary hyperalgesia enhances nociception only to mechanical stimuli adjacent to the area of incision. This is likely caused by the sensitization of the central nervous system [41]. It is believed that, in humans, primary and secondary hyperalgesia develops after operation involving both peripheral and central sensitization [38]. Ilkjaer et al. [42] also reported that mechanical hyperalgesia can easily be detected 24 hours after a total abdominal hysterectomy.

Prostaglandin released from the damaged cells at the wound is a potent proinflammatory molecule for inflammatory nociceptive sensitization [43]. N-methyl-d-aspartate can be activated by repeated noxious stimulations that contribute to the development and maintenance of sensitization [44]. Electroacupuncture can attenuate the release of prostaglandin [45] and modulate the expression of N-methyl-d-aspartate [46] in animal models, indicating that it may inhibit hyperalgesia at both the peripheral and spinal levels. We postulate that the use of auricular TENS may achieve similar effects and hence reduce movement-evoked pain in our postoperative patients. This view may warrant further investigation.

The present study randomly assigned patients into three groups to receive true auricular TENS, sham TENS, or no stimulation (control). By comparing the results of the sham TENS group with those of the control group, we may estimate the extent of the placebo effect of auricular TENS. By comparing the results of the true TENS group with those of the sham TENS group, we may estimate the genuine analgesic effect produced by auricular TENS.

The patients in the sham TENS group received stimulation to inappropriate auricular points and still reported a maximal reduction of 14.0% in VAS-rest, 4.7% in VAS-huff, and 5.1% in VAS-cough. In contrast, almost no pain reduction was reported in the control group. Greater analgesic effects tended to be observed in the sham TENS group than in the control group, but the post hoc test did not find any significant differences between the two groups. The analgesic effect observed in the sham TENS group could have been due to either the placebo effect or the fact that intense sham auricular TENS applied on inappropriate auricular points may produce certain nonspecific physiological effects [47], which triggers the Diffuse Noxious Inhibitory Control.

Patients in the true TENS group and the sham TENS group received exactly the same intervention except the sites of stimulation. Only the true TENS group received auricular TENS at the therapeutic auricular points resulted in better analgesic effects than that of the sham TENS group, followed by the control group. The post hoc test showed that significant group difference came mainly from the comparison between the true TENS group and the control group. It indicates that better analgesic effect observed in the true TENS group should not be just a placebo effect and such an analgesic effect appeared to be point specific.

Despite the fact that auricular TENS could significantly reduce the pain felt during huffing and coughing, it had no effect on the performance of PEFR. We have conducted a power analysis for our study; they were greater than 0.8 for the between-group comparisons made in the three VAS measurements (i.e., VAS-rest, VAS-huff, and VAS-cough), but the power was low for the outcome on PERF (0.35) that may probably be due to the small sample size and high individual differences observed in the present study. Previous studies have reported that TENS significantly improved pulmonary function after cardiac and thoracic surgery but not after abdominal surgery [37]. The functional integrity of the abdominal muscles is considered to be very important in the process of generating coughs [48]. Unlike patients who have undergone cardiac or thoracic surgery, whose abdominal muscles remain intact, patients experience a loss of muscle integrity after an abdominal surgery, which probably accounts for their lack of improvement in forced expiration. Clinical observations and previous study [49] suggest that huffing or coughing efforts are inhibited by intense postoperative pain; however, such efforts at forced expiration may not necessarily improve following relief from such pain. In fact, the performance of huffing or coughing is influenced by various factors other than pain; for example, lung volumes, sensitivity of airway reflexes, muscle biomechanics, medications, and the patient's state of mind [50].

Gilron et al. [49] reported that the pain experienced during coughing was significantly and negatively correlated with PEFR in patients after abdominal hysterectomy. We found that the mean VAS-cough scores in the sham TENS group remained quite stable throughout the study (Figure 5). No significant change was therefore observed in the performance of PEFR in this group of patients.

Contrary to the PEFR performance observed in the two treatment groups, the PEFR performance in the control group declined significantly across the study period. This significant drop in PEFR may be due to the uncontrolled pain experienced during the forced expiration maneuver. The mean VAS-cough score of the control group was higher than those of the two treatment groups (Figure 5). The significant drop in PEFR in the control group may be due to the avoidance of forced expiration maneuvers in this group of patients.

Compared to the use of conventional TENS with electrodes placed adjacent to the incision sites, auricular TENS has an advantage because it maintains the integrity of wound dressing and hence reduces the risk of wound infections. For postoperative patients in critical condition, intravenous lines are commonly found on the limbs, leads of electrocardiogram over the chest, and bulky dressings on the abdomen. Auricular TENS is a favorable analgesic means as it would not interfere with the surrounding monitoring system. Auricular electroacupuncture is also a treatment option for postoperative pain, but it is an invasive procedure that involves the use of auricular acupuncture needles as electrodes. Needling acupuncture points on the auricle may lead to complications such as perichondritis [51]. Since auricular stimulation delivered through surface electrode can be as effective as needle acupuncture [52], the use of auricular TENS would seem to be preferable.

Patients aged above 60 were excluded from the present study because elderly are known to under report their pain [53]. Moreover, Chinese elderly commonly lack the knowledge of pain management. They are reluctant to express their pain because of the worry that analgesic treatment will affect wound healing [54]. Adopting this exclusion criteria may limit the generalizability of the study result to older age population. However, a recent surveillance of hysterectomy indicated that the incidents of hysterectomy were highest among women aged 40–44 and the incidents above the age of 55 were quite low [55]. Our study focused only on the immediate analgesic effect of auricular TENS. We standardized the amount and timing of the oral medication and intramuscular injected analgesics consumed by all of the subjects. A patient-controlled analgesia pump can be used in future study, so that the effectiveness of repeated applications of auricular TENS over a few days can be reflected by the total consumption of analgesics in that period of time.

To conclude, a single session of auricular TENS applied to therapeutic auricular points significantly reduced the resting pain (VAS-rest) and movement-evoked pain (VAS-huff, VAS-cough) experienced by patients after a total abdominal hysterectomy. The reduction in pain outlasted the treatment for at least 30 minutes. The analgesic effects of auricular TENS appear to be point specific, and cannot be explained by the placebo effect alone. However, auricular TENS did not produce a significant improvement in the performance of PEFR.

## Figures and Tables

**Table 1:** Demographic characteristics and baseline data of the three study groups.

Group	True TENS (*n* = 16)	Sham TENS (*n* = 16)	Control (*n* = 16)	*P* value (between group)
Age (years)	45.31 ± 2.68	45.88 ± 3.91	44.63 ± 4.92	.671
Body mass index (kg/m^2^)	24.43 ± 3.91	22.49 ± 3.90	25.28 ± 4.13	.138
Duration of operation (minutes)	112.06 ± 36.96	98.75 ± 19.45	115.44 ± 36.15	.303
Duration of general anesthesia (minutes)	136.06 ± 37.96	118.56 ± 20.81	139.19 ± 37.38	.175
Type of incision (T: Transverse/M: Midline)				
T	9 (56)	12 (75)	11 (69)	.519
M	7 (44)	4 (25)	5 (31)	
Intraoperation fentanyl consumption (*μ*g/kg)	68.75 ± 39.26	68.75 ± 37.08	71.88 ± 46.44	.970
Total morphine consumption (mg)	8.59 ± 4.11	8.59 ± 3.48	9.06 ± 4.25	.928
Postoperation pethidine consumption (mg)	75.00 ± 47.43	64.06 ± 45.62	87.50 ± 53.23	.405
Postoperation dologesic consumption (tablets)	1.31 ± 1.25	1.50 ± 1.15	0.88 ± 0.96	.284
Time of intervention (hours after operation)	24.40 ± 0.72	24.70 ± 0.96	25.08 ± 1.20	.156
Analgesic-free period (hours)	7.54 ± 6.60	5.66 ± 4.99	5.63 ± 4.41	.527

Values were expressed as mean ± standard deviation.

The type of incision was expressed as a number (percentage).

Total morphine consumption: morphine consumed during the operation and in the recovery room.
